# Production of free monounsaturated fatty acids by metabolically engineered *Escherichia coli*

**DOI:** 10.1186/1754-6834-7-59

**Published:** 2014-04-10

**Authors:** Yujin Cao, Wei Liu, Xin Xu, Haibo Zhang, Jiming Wang, Mo Xian

**Affiliations:** 1 CAS Key Laboratory of Bio-based Materials, Qingdao Institute of Bioenergy and Bioprocess Technology, Chinese Academy of Sciences, Qingdao, China

**Keywords:** Free monounsaturated fatty acids, Thioesterase, Fatty acid desaturase, acyl-CoA synthetase, acetyl-CoA carboxylase

## Abstract

**Background:**

Monounsaturated fatty acids (MUFAs) are the best components for biodiesel when considering the low temperature fluidity and oxidative stability. However, biodiesel derived from vegetable oils or microbial lipids always consists of significant amounts of polyunsaturated and saturated fatty acids (SFAs) alkyl esters, which hampers its practical applications. Therefore, the fatty acid composition should be modified to increase MUFA contents as well as enhancing oil and lipid production.

**Results:**

The model microorganism *Escherichia coli* was engineered to produce free MUFAs. The fatty acyl-ACP thioesterase (AtFatA) and fatty acid desaturase (SSI2) from *Arabidopsis thaliana* were heterologously expressed in *E. coli* BL21 star(DE3) to specifically release free unsaturated fatty acids (UFAs) and convert SFAs to UFAs. In addition, the endogenous *fadD* gene (encoding acyl-CoA synthetase) was disrupted to block fatty acid catabolism while the native acetyl-CoA carboxylase (ACCase) was overexpressed to increase the malonyl coenzyme A (malonyl-CoA) pool and boost fatty acid biosynthesis. The finally engineered strain BL21ΔfadD/pE-AtFatAssi2&pA-acc produced 82.6 mg/L free fatty acids (FFAs) under shake-flask conditions and FFAs yield on glucose reached about 3.3% of the theoretical yield. Two types of MUFAs, palmitoleate (16:1Δ9) and *cis*-vaccenate (18:1Δ11) made up more than 75% of the FFA profiles. Fed-batch fermentation of this strain further enhanced FFAs production to a titer of 1.27 g/L without affecting fatty acid compositions.

**Conclusions:**

This study demonstrated the possibility to regulate fatty acid composition by using metabolic engineering approaches. FFAs produced by the recombinant *E. coli* strain consisted of high-level MUFAs and biodiesel manufactured from these fatty acids would be more suitable for current diesel engines.

## Background

Biodiesel is a mixture of fatty-acid alkyl esters obtained by the transesterification of triglycerides (in most cases, vegetable oils and animal fats) with methanol or ethanol. It is a renewable alternative with the potential to replace the petroleum-based diesel. The properties of a biodiesel fuel including cetane number, density, viscosity, flash point, oxidative stability and cold-filter plugging point, are determined by the structure of its component alcohols and fatty acids [1], whereas the properties of an individual fatty acid depend on the chain length, the occurrence of double bonds and branch chains [2]. For instance, the calorific value of biodiesel increases along with fatty-acid chain length, but the low temperature fluidity decreases with chain length. The longer the chain, the greater the viscosity, and *cis* double bonds also lower the viscosity [3]. According to a report from the US Department of Energy, the perfect biodiesel would be made only from monounsaturated fatty acids (MUFAs) [4]. An ideal biodiesel composition should have fewer polyunsaturated and saturated fatty acids (SFAs) [5]. High levels of polyunsaturated fatty acids (PUFAs) would negatively impact the oxidative stability and increase nitrogen oxide exhaust emissions, which do not suit diesel engines [6,7]. On the contrary, biodiesel derived from SFAs would have good oxidative stability, but poor fuel properties at low temperatures, which is a disadvantage in winter operation [8].

At present, commercially available biodiesel is mostly produced from vegetable oils. Unfortunately, fatty acid composition varies greatly between different vegetable oils. As shown in Table 1, many of these vegetable oils contain a high level of linoleate (18:2Δ9,12) whereas others contain high amounts of palmitate (16:0). Therefore, the properties of vegetable oil-based biodiesel always need to be adjusted by certain additives to enhance its utilization potential. In addition, the excessive consumption of vegetable oils is a threat to global food security. In recent years, the use of microbial lipids, which can be obtained by fermenting non-food feedstocks, to synthesize biodiesel is attracting increasing attention. A number of oleaginous microorganisms such as microalgae [9], yeasts [10] and fungi [11], have been tested for their capability for lipid accumulation. As a reference microorganism, *Escherichia coli* possesses many advantages over other microorganisms, for example, a clear genetic background, the convenience of its ability to be genetically modified, and good growth properties with low nutrient requirements [12,13]. It has been widely studied for production of fatty acids [14,15], or directly biodiesel [16,17], and shows a promising prospect for industrial application. Previous studies have demonstrated that thioesterase expression is a key step to produce free fatty acids (FFAs) in *E. coli*. Heterologous expression of different thioesterases would lead to accumulation of significant amounts of FFAs [18]. Blocking fatty acid consumption is also critical for FFA production in *E. coli*. A promising alternative route to produce FFAs would involve reversing β-oxidation so that it operates anabolically to the fatty-acid biosynthesis direction [19]. The irreversible synthesis of malonyl-CoA catalyzed by acetyl-CoA carboxylase is another key regulatory point for fatty acid production. Overexpression of this enzyme could increase the final titer of FFAs in the engineered strain [20]. Compared with vegetable oils and microalgal lipids, biodiesel manufactured by *E. coli* does not contain PUFAs. However, SFAs make up the majority of *E. coli* lipids [21]. Modifying the fatty acid compositions of *E. coli* is critical to promote its practical application in the biodiesel industry.

**Table 1:** Fatty acid compositions of several typical vegetable oils

**Vegetable oils**	**12:0**	**14:0**	**16:0**	**16:1**	**18:0**	**18:1**	**18:2**	**18:3**	**22:1**	**References**
Soybean oil	NR	NR	11%	NR	4%	23.4%	53.2%	7.8%	Traces	[22]
Corn oil	NR	NR	10.3%	NR	1.0%	30.3%	58.0%	0.4%	NR	[23]
Rapeseed oil	NR	NR	5.51%	NR	2.17%	58.33%	19.89%	9.13%	4.59%	[24]
Palm oil	0.1%	1.0%	42.8%	NR	4.5%	40.5%	10.1%	0.2%	NR	[25]
Jatropha oil	NR	NR	15.32%	1.33%	4.06%	35.38%	43.34%	NR	NR	[26]

NR: Not reported.

12:0, laurate; 14:0, myristate; 16:0, palmitate; 16:1, palmitoleate; 18:1, oleate; 18:2, linoleate; 18:3, linolenate; 22:1, erucate.

**Figure 1:**
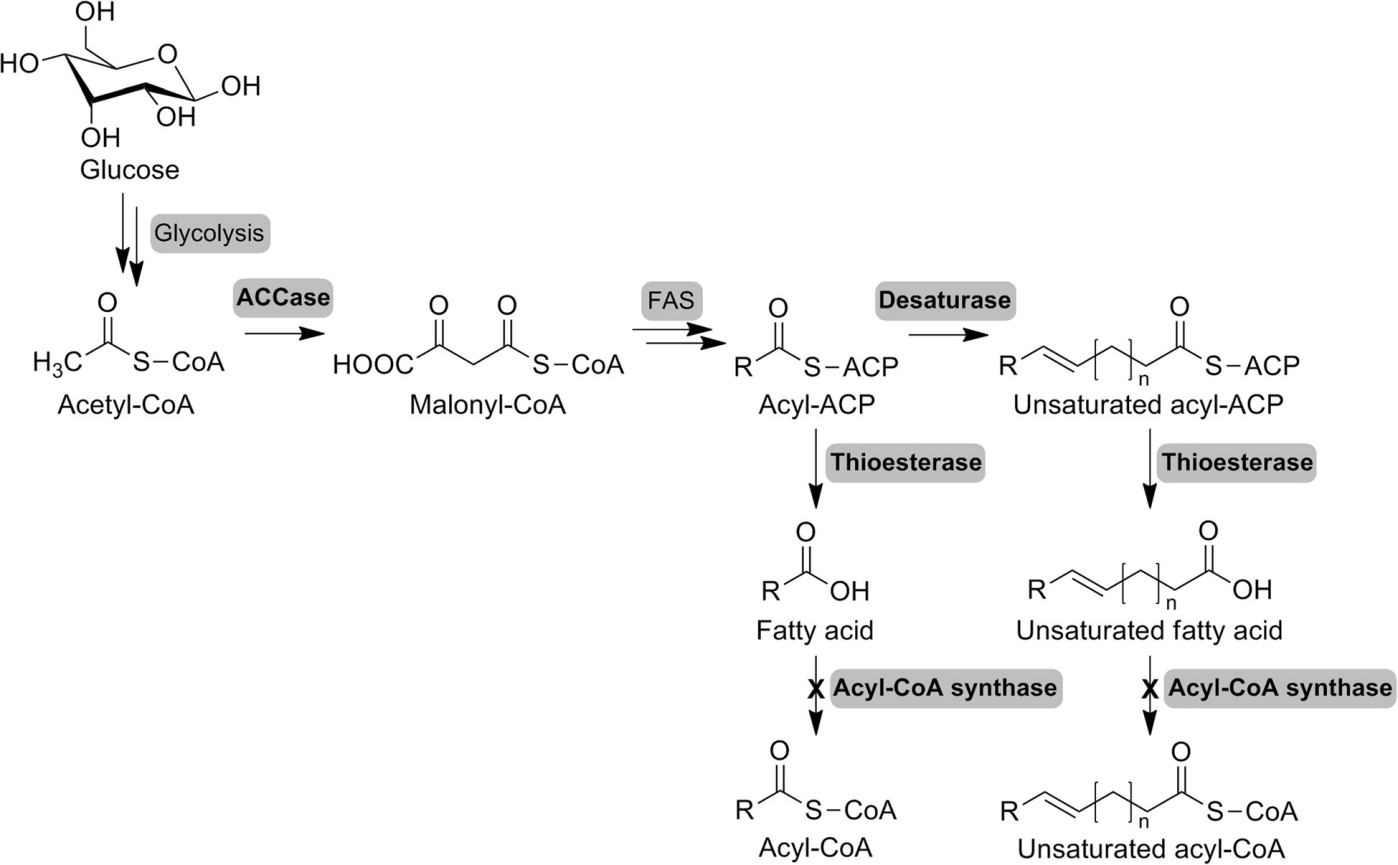
**Biosynthetic pathway of free monounsaturated fatty acid in this study.** ACCase, acetyl-CoA carboxylase; FAS, fatty acid synthase.

In this study, four distinct genetic alterations targeted at free MUFA production were introduced into the host strain *E. coli* BL21 star(DE3) (Figure 1), including heterologous alterations expressing a fatty acyl carrier protein (ACP) thioesterase to render *E. coli* capable of producing FFAs; introducing a fatty-acid desaturase to convert SFAs to unsaturated fatty acids (UFAs); knockout of the endogenous *fadD* gene, which encodes the acyl-CoA synthetase, to block native fatty-acid catabolism; and further overexpression of the acetyl-CoA carboxylase to increase the malonyl-CoA pool and boost fatty-acid biosynthesis. The finally engineered strain was cultured under fed-batch conditions to evaluate its ability to produce MUFAs.

## Results and discussion

### Production of free unsaturated fatty acids by a specific thioesterase

As a model organism to study fatty-acid biosynthesis, *E. coli* has a type II FAS (FAS II) system, which is found in most bacteria and plants [27]. In this biosynthetic system, fatty acids are bound to ACP and catalyzed by a series of discrete, mono-functional enzymes [28]. However, the fatty acids in *E. coli* are only used to synthesize membrane phospholipids and lipid A. Few FFAs are produced by wild-type *E. coli* strains. Fatty acid thioesterase (Fat) is an enzyme that cleaves the fatty acid thioester bond to coenzyme A (CoA) or ACP. The *E. coli* genome only carries two acyl-CoA thioesterases (*tesA* and *tesB*), which catalyze the hydrolytic cleavage of fatty acyl-CoA thioesters and function in the process of fatty-acid degradation [29]. Although these two enzymes can also cleave the bonds of fatty acyl-ACP thioesters, the catalytic efficiency is much lower than acyl-CoA esters of the same length [30].

Acyl-ACP thioesterases can hydrolyze the thioester bond between the acyl moiety and ACP. These enzymes play an essential role in chain termination during *de novo* fatty-acid synthesis in higher plants [31]. Expression of plant acyl-ACP thioesterases can result in dramatic changes in the fatty-acid profiles both in *E. coli* cell membrane and culture supernatant [32-34]. According to the substrate specificity, there are two types of acyl-ACP thioesterases, FatA and FatB. Substrate specificity of these isoforms depends on the chain length and saturation level of fatty acids. The highest activity of FatA is with unsaturated acyl-ACP whereas FatB prefers palmitoyl (16:0)-ACP as its optimal substrate [35]. The *Arabidopsis thaliana AtFatA* gene encodes a thioesterase with preference towards oleoyl (18:1Δ9)-ACP. The catalytic efficiencies of AtFatA are always several-fold higher for the monoenes than their saturated counterparts [36].

**Figure 2:**
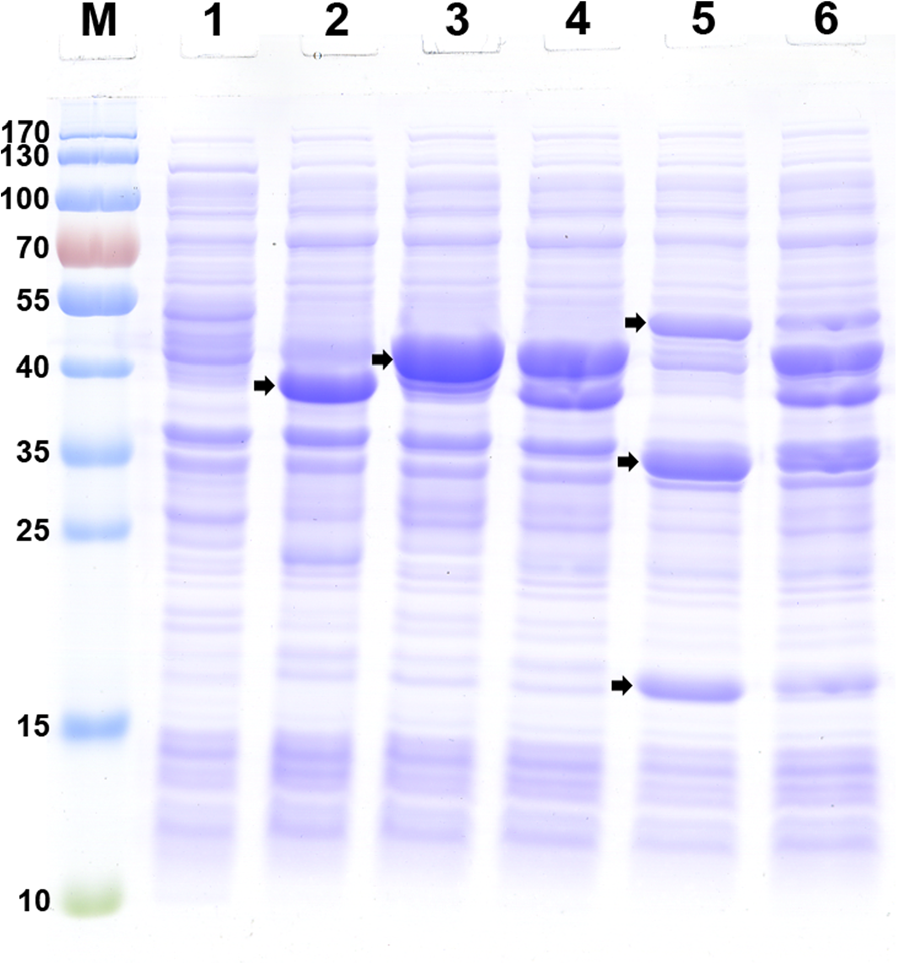
**SDS-PAGE analysis of different recombinant proteins expressed in *E. coli*.** Recombinant protein expression was induced using 0.1 mM isopropyl-ß-D-thiogalactopyranoside (IPTG) for a cultivation time of 4 h at 37°C. Lane M, prestained protein molecular weight marker; lane 1, control strain harboring pET30a; lane 2, crude cells extracts from strain BL21/pA-AtFatA; lane 3, crude cells extracts from strain BL21/pE-ssi2; lane 4, crude cells extracts from strain BL21/pE-AtFatAssi2; lane 5, crude cells extracts from strain BL21/pA-acc; lane 6, crude cells extracts from recombinant strain harboring both pE-AtFatA and pA-acc. The bands corresponding to the individual proteins were indicated by an arrow.

In order to produce free UFAs, the thioesterase AtFatA was heterologously expressed in *E. coli* BL21 star(DE3). The coding region of this gene was cloned into the pACYCduet-1 expression vector under T7 promoter, resulting pA-AtFatA. The recombinant constructs were checked by restriction enzyme digestion and DNA sequencing. To verify the expression level of the recombinant protein, *E. coli* competent cells were transformed by the expression vector pA-AtFatA and grown in liquid LB medium to an optical density (OD)_600_ of 0.6 followed by induction using 0.1 mM isopropyl-ß-D-thiogalactopyranoside (IPTG). Figure 2 showed the gel electrophoresis patterns of samples from different recombinant strains analyzed with coomassie brilliant blue staining (to visualize all proteins). Lane 2 was the cell lysates of strain BL21/pA-AtFatA. It could be found that AtFatA was soluble expressed in *E. coli* (corresponding to the band of molecular weight 40.8 kDa).

**Figure 3:**
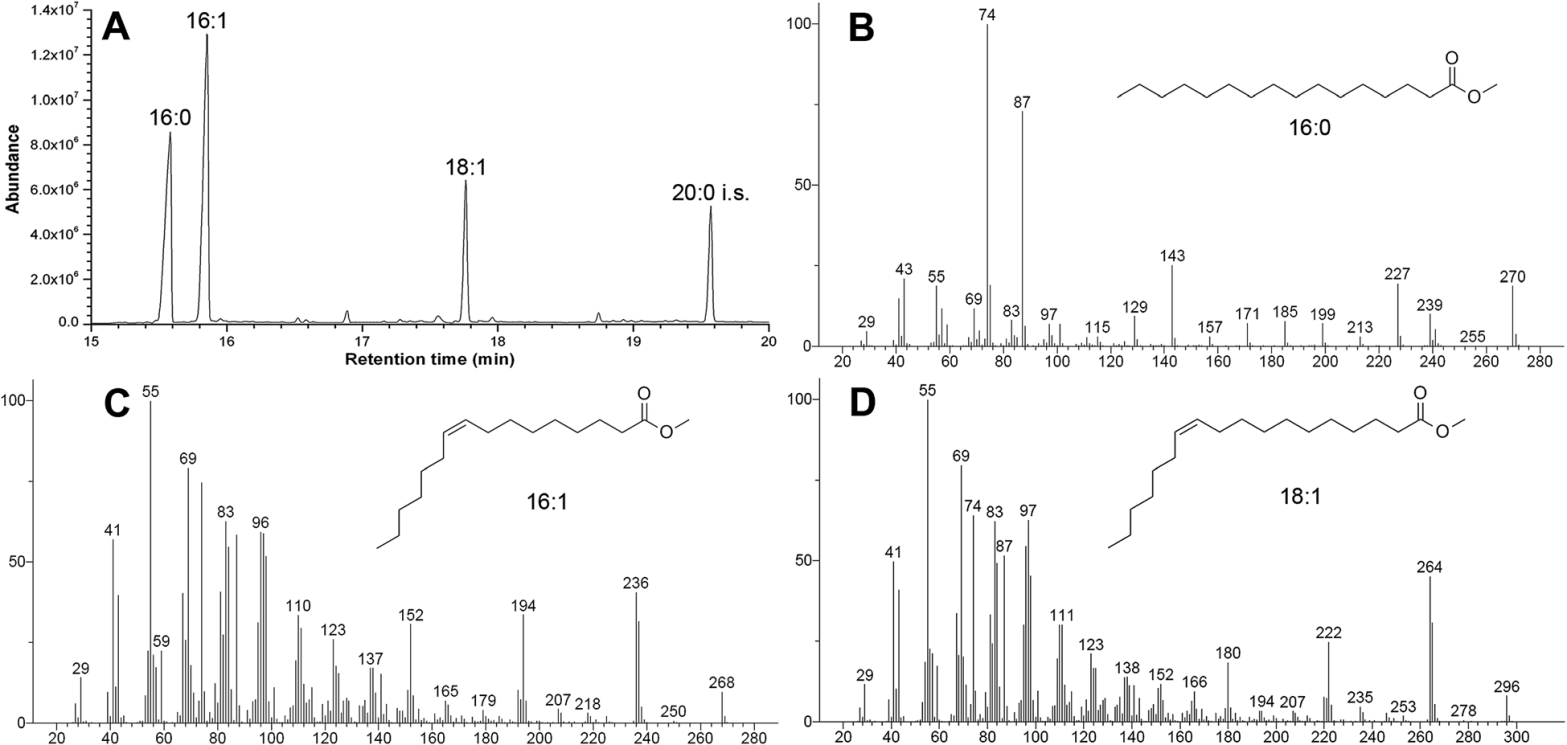
**Identification of free fatty-acid compositions of the AtFatA-expressing strain by gas chromatography-mass spectrometry (GC-MS). (A)** A total ion chromatogram (TIC) of free fatty-acid methyl esters from *E. coli* BL21 star(DE3) harboring pA-AtFatA after being induced for 16 h. **(B)** Mass spectrum of palmitate methyl ester (16:0); **(C)** mass spectrum of palmitoleate methyl ester (16:1Δ9); **(D)** mass spectrum of *cis*-vaccenate methyl ester (18:1Δ11). The structures were matched by searching a standard NIST library. The molecular ion peaks of these fatty-acid methyl esters are marked with circles.

To evaluate the effects of AtFatA on FFA production, the recombinant strain BL21/pA-AtFatA was cultured using M9 minimal medium under shake-flask conditions. After being induced for 16 h, FFAs were extracted, derivatized and identified by gas chromatography-mass spectrometry (GC-MS) analysis. As shown in Figure 3, three types of fatty acid, that is, palmitoleate (16:1Δ9), palmitate (16:0) and *cis*-vaccenate (18:1Δ11) made up the major components of the AtFatA-expressing strain. In addition, trace amounts of myristate, *cis*-methylene-9,10-hexadecanoate and *cis*-methylene-11,12-octadecanoate were also detected. The two types of cyclopropane fatty acids (CFAs) were formed by modification of the double bond of UFAs in the phospholipids, which was catalyzed by CFA synthase [37,38]. CFAs should only exist in the cellular membrane and the appearance of CFAs might be due to the fact that some of them were extracted along with FFAs by ethyl acetate. To determine the contents of these fatty acids, the FFA profiles were further analyzed by gas chromatography (GC) coupled with a flame ionization detector (FID). The amount of each fatty acid was computed from the area under the corresponding peak. The recombinant strain carrying pA-AtFatA overproduced 16:1 and 18:1 fatty acid. Free palmitoleate and *cis*-vaccenate levels of this strain were 45.6% and 21.5%, respectively, and the ratio of UFA to SFA reached 2:1. The total FFA production was about 23.4 mg/L and cell density of the final culture broth reached an OD_600_ of 3.1. Although the palmitoyl-ACP pool was much larger in *E. coli*[39,40], palmitoleate levels of the FFA profiles in the strain heterologously expressing AtFatA were much greater than palmitate. This phenomenon can be interpreted by the fact that AtFatA preferred unsaturated acyl-ACP as its substrates rather than saturated acyl-ACP. On the other hand, oleate (18:1Δ9) is not the major constituent of *E. coli* fatty acids and *cis*-vaccenate seems not to be a good substrate for this enzyme. Thus, expression of AtFatA individually does not obviously increase the *cis*-vaccenate level. Compared with previous studies using thioesterases to produce FFAs in *E. coli*[41-43], the contents of free MUFAs were greatly enhanced. However, the total FFA production was much lower. The low FFA titer achieved in this study might be due to the less active AtFatA. The acyl-CoA thioesterases of bacterial origin seemed to be more effective for FFA production.

### Increasing unsaturated fatty-acid content by a fatty-acid desaturase

The UFA biosynthesis pathway of *E. coli* is different from many other bacteria and higher plants. Wild-type *E. coli* does not encode any fatty-acid desaturase. It employs an anaerobic pathway to synthesize UFAs. Fatty-acid desaturases are enzymes that introduce double bonds into the hydrocarbon chains of fatty acids. They are important to the maintenance of the proper structure and function of biological membranes [44]. Plant fatty-acid desaturases can use acyl chains attached to *E. coli* ACP as substrates and several of these enzymes have been characterized by previous researchers [45-47]. The fatty-acid desaturase SSI2 from *Arabidopsis* catalyzes the conversion of stearoyl (18:0)-ACP to oleoyl-ACP through the eukaryotic pathway of plant lipid biosynthesis [48]. Thus, we coexpressed this enzyme with the thioesterase AtFatA in *E. coli*. Figure 2, lane 3 (corresponding to the band of molecular weight 45.6 kDa) and lane 4 shows the electrophoresis spectrums of strain BL21/pE-ssi2 and BL21/pE-AtFatAssi2. Both of the two enzymes were successfully expressed in *E. coli*.

In order to investigate the effects of SSI2 on FFA compositions, recombinant *E. coli* strains harboring pE-AtFatAssi2 were cultured under the same conditions for 16 h as discussed above. Samples were taken from the culture broth and fatty acids were analyzed by GC. The fatty-acid profiles of strain BL21/pA-AtFatA and BL21/pE-AtFatAssi2 were compared in Table 2. We found that the introduction of the SSI2 desaturase did not affect total FFA production of the recombinant strain. However, the contents of the two UFAs increased when compared with the AtFatA solely expressed strain. The ratio of UFA to SFA reached about 3.4 in this engineered strain. As the optimal substrate stearoyl-ACP for the SSI2 desaturase was not present as the major component in *E. coli*, this enzyme used palmitoyl-ACP as an alternative substrate to produce palmitoleate. The overproduced palmitoleate was further elongated by *fabF* (encoding *E. coli* β-ketoacyl-ACP synthase II) [49], resulting in an enhanced *cis*-vaccenate level. Therefore, the contents of both of the two UFAs were improved to some extent.

**Table 2:** Effects of the fatty-acid desaturase SSI2 on free fatty-acid compositions

**Strains**	**OD**_**600**_	**Free fatty acids (mg/L)**				
		**16:1**	**16:0**	**18:1**	**Total**	**UFA:SFA**
BL21/pA-AtFatA	3.27 ± 0.40	10.7 ± 2.6	7.7 ± 1.5	5.0 ± 1.1	23.4 ± 4.6	2.0:1
BL21/pE-AtFatAssi2	2.95 ± 0.31	10.6 ± 2.2	5.0 ± 0.9	6.2 ± 1.0	21.8 ± 3.4	3.4:1

OD, optical density; UFA, unsaturated fatty acids; SFA, saturated fatty acids. The values represent mean ± SD.

### Inactivation of the native fatty-acid catabolic pathway

*E. coli* can utilize long-chain fatty acids as its sole carbon and energy source, which might inhibit FFA production. Along with the development of modern molecular biology, the fatty-acid uptake mechanism has been largely resolved. *E. coli* has evolved a highly regulated fatty-acid transport system comprised of the outer membrane protein FadL, the periplasmic protein Tsp and the inner membrane-associated enzyme FadD [50]. Exogenous fatty acids could bind to the FadL protein with high affinity. With the help of the Tsp protein, fatty acids are transferred across the double membrane and finally activated by acyl-CoA synthetase, which is encoded by *fadD*[51]. Among the three proteins, FadD is shown to catalyze the rate-limiting step for fatty-acid utilization. *E. coli fadD* mutants could accumulate FFAs released from membrane lipids in the stationary phase [52]. To further increase the total FFA content, we disrupted the *fadD* gene in the *E. coli* BL21 star(DE3) chromosome using the Red recombination method, resulting strain BL21ΔfadD. Successful gene disruptions were confirmed by PCR amplification (see Additional file 1: Figure S1). Liquid growth tests on M9 minimal medium supplemented with palmitate as the sole carbon source further proved that the fatty-acid catabolism-blocked strain, BL21ΔfadD, grew much more poorly than its parent strain.

**Figure 4:**
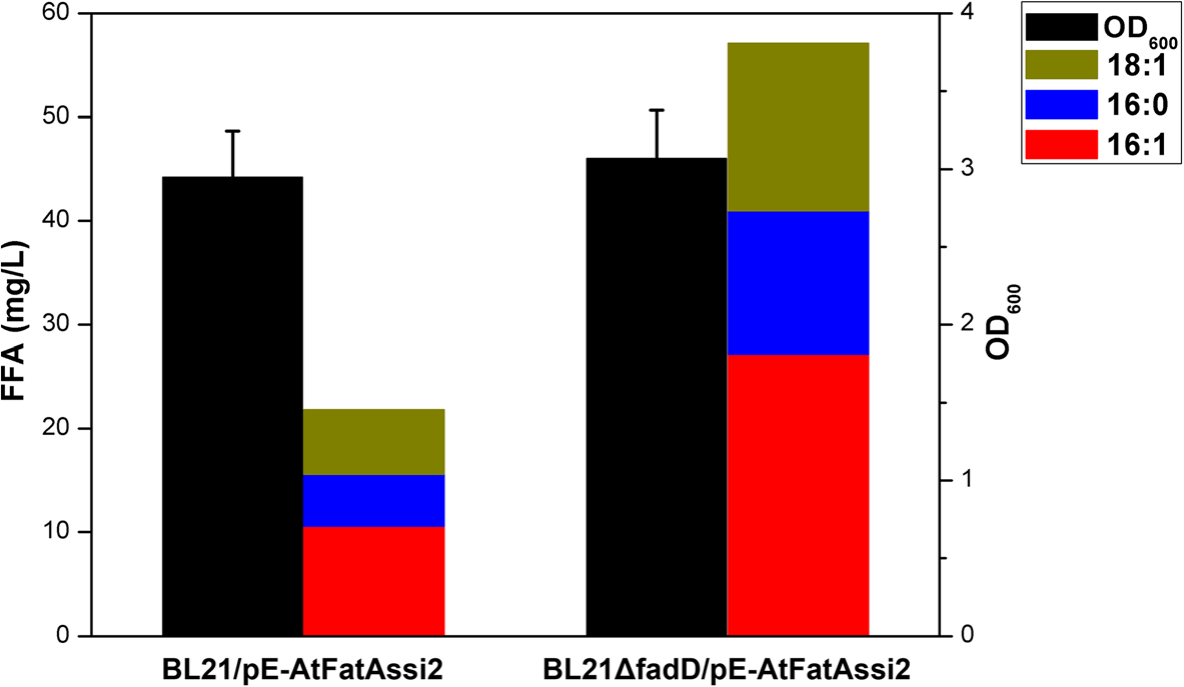
**Effects of *fadD *knockout on free fatty-acid production.** BL21/pE-AtFatAssi2, strain BL21 star(DE3) expressing *Arabidopsis* thioesterase AtFatA and fatty acid desaturase SSI2; BL21ΔfadD/pE-AtFatAssi2, knockout of native *fadD* while expressing AtFatA and SSI2; 16:1, palmitoleate; 16:0, palmitate; 18:1, *cis*-vaccenate. Data were obtained after each strain was induced for 16 h in liquid M9 minimal medium supplemented with 1 mM MgSO_4_ and 20 g/L glucose. FFA, free fatty acid; OD, optical density.

With the aim to evaluate the effects of *fadD* disruption on free UFA production, the recombinant plasmid pE-AtFatAssi2 was transformed into strain BL21ΔfadD. The resulting strain BL21ΔfadD/pE-AtFatAssi2 was cultured under shake-flask conditions. Cell density and different types of FFA production were monitored over the course of the fermentation. As shown in Figure 4, the total FFA production was greatly enhanced in the *fadD* mutant strain. After being induced for 16 h, the *fadD* mutant strain harboring pE-AtFatAssi2 accumulated 57.1 mg/L of FFAs, which was 2.4-fold that of strain BL21/pE-AtFatAssi2, and the cell growth was comparable between the two strains at this time point. Compared with previous reports [14,20], disruption of the *fadD* gene showed similar effects on FFA production. Also, the ratio of UFAs remained stable between the two strains. These results demonstrated that knockout of the acyl-CoA synthetase enhanced FFA production without affecting the fatty-acid compositions.

### Boosting fatty-acid production by overexpressing native *E. coli* acetyl-CoA carboxylase

The above strains successfully accumulated considerable amounts of free MUFAs in the culture broth. However, this level of production was still far beyond industrial applications. In biological systems, malonyl-CoA is the direct precursor for fatty-acid biosynthesis. However, *E. coli* only maintains a very low level of malonyl-CoA for natural anabolism [53]. The insufficient supply of intracellular malonyl-CoA hampers high-level FFA production. Acetyl-CoA carboxylase (ACCase), which catalyzes the irreversible carboxylation of acetyl-CoA, is the only producer of malonyl-CoA [54]. Previous studies have shown that overexpression of ACCase from different origins increased the pool of malonyl-CoA and the rate of fatty-acid synthesis [55,56]. Here, we overexpressed native *E. coli* ACCase together with *AtFatA* and *ssi2* to further boost free MUFA production.

**Figure 5:**
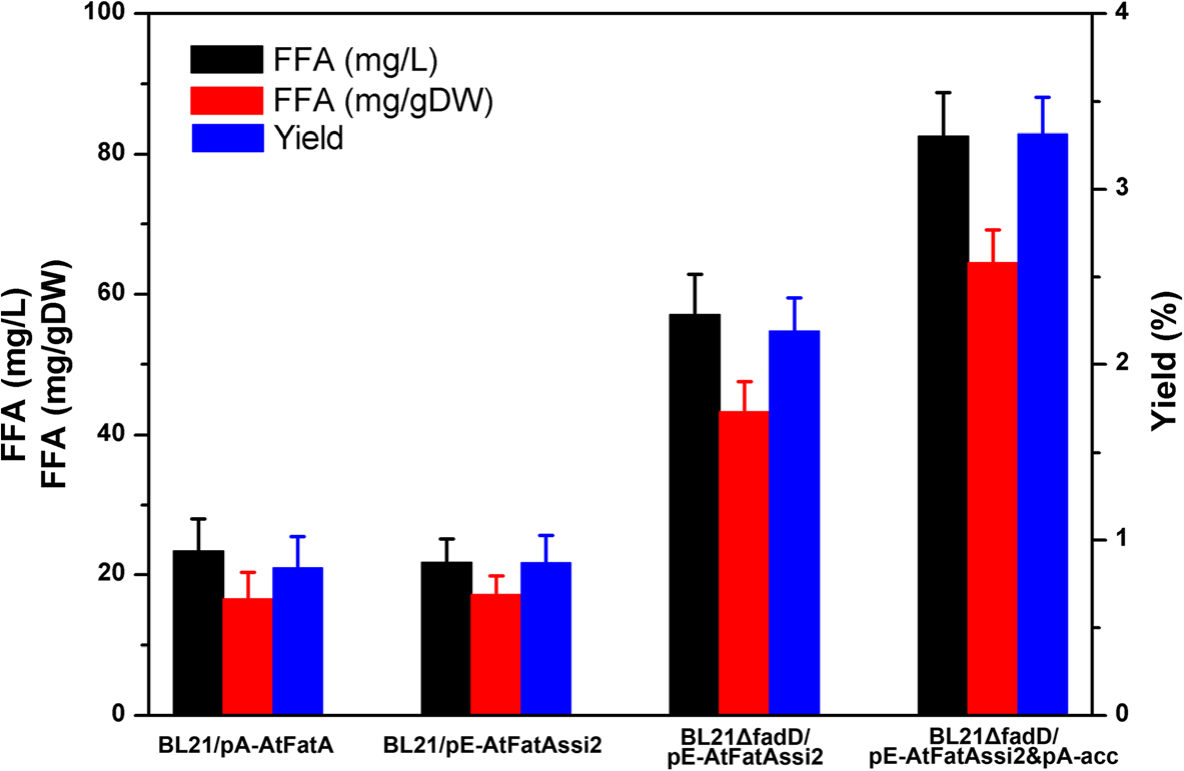
**Comparison of free fatty-acid production and the yields on glucose of several different strains.** BL21/pA-AtFatA, strain BL21 star(DE3) expressing *Arabidopsis* thioesterase; BL21/pE-AtFatAssi2, strain BL21 star(DE3) expressing *Arabidopsis* thioesterase and fatty-acid desaturase; BL21ΔfadD/pE-AtFatAssi2, knockout of native *fadD* while expressing AtFatA and SSI2; BL21ΔfadD/pE-AtFatAssi2&pA-acc, further overexpression native of *E. coli* acetyl-CoA carboxylase. Data were obtained after each strain was induced for 16 h in liquid M9 minimal medium supplemented with 1 mM MgSO_4_ and 20 g/L glucose except for the recombinant strain BL21ΔfadD/pE-AtFatAssi2&pA-acc (12 h). FFA, free fatty acid.

Figure 2, line 5 shows the gel electrophoresis pattern of the recombinant strain carrying pA-acc and line 6 is the recombinant strain carrying both pE-AtFatAssi2 and pA-acc. In all cases, the four subunits of ACCase were clearly expressed and we noted distinct bands of the expected size from bacterial extracts when compared with the control strain. Then strain BL21ΔfadD/pE-AtFatAssi2&pA-acc was cultured using M9 minimal medium under shake-flask conditions. FFA compositions of different engineered *E. coli* strains are presented in Table 3 and the corresponding GC chromatograms are provided (see Additional file 2: Figure S2). The amounts of FFA accumulated in the fermentation broth, the productivity of FFAs per cell dry weight (DW) and the yields of FFA on glucose of different recombinant strains were calculated and are shown in Figure 5. It could be seen that the ACCase overexpression strain produced more FFAs than other strains. The final titer of FFAs reached 82.6 mg/L, which was 3.5-fold that of the AtFatA solely expressed strain and 1.4-fold that of the *fadD* mutant strain. The productivity of FFAs per cell DW of the ACCase overexpression strain was 64.5 mg/g DW. The yield of FFAs on glucose of this strain also reached about 3.3% of the theoretical yield (the theoretical yield of 35.6% was formula):

4Glucose→8Acetyl‒CoA→Palmitate

**Table 3:** **Free fatty-acid compositions of different metabolically engineered *E. coli *** **strains**

**Strains**	**Free fatty acids (%)**					
	**14:0**	**16:1**	**16:0**	17:0c^*^	**18:1**	19:0c^**^
BL21/pA-AtFatA	1.8	44.6	32.1	0.5	20.8	0.2
BL21/pE-AtFatAssi2	1.9	47.4	22.2	0.5	27.7	0.3
BL21ΔfadD/pE-AtFatAssi2	1.5	47.4	22.8	0.2	27.9	0.2
BL21ΔfadD/pE-AtFatAssi2&pA-acc	1.8	47.5	22.6	0.2	27.8	0.1

^*^cis-methylene-9,10-hexadecanoate; ^**^cis-methylene-11,12-octadecanoate.

This yield was far from the theoretical limits and much lower than many previous reports [20,43,57]. Considering the relatively low catalytic activity of the thioesterase AtFatA, we can expect to achieve a high titer of FFAs by improving the efficiency of this enzyme.

In addition, the recombinant strain BL21ΔfadD/pE-AtFatAssi2&pA-acc accumulated FFAs much faster. FFA concentrations of the ACCase overexpression strain reached the maximum level after being induced for 12 h, whereas the other strains required about 16 h to achieve the maximum titer. Fatty-acid composition analysis showed that overexpression of ACCase did not affect the FFA constituents either. MUFAs were the predominant components of the total FFA profiles and the ratio of UFA to SFA was above 3:1.

### Fed-batch fermentation

**Figure 6:**
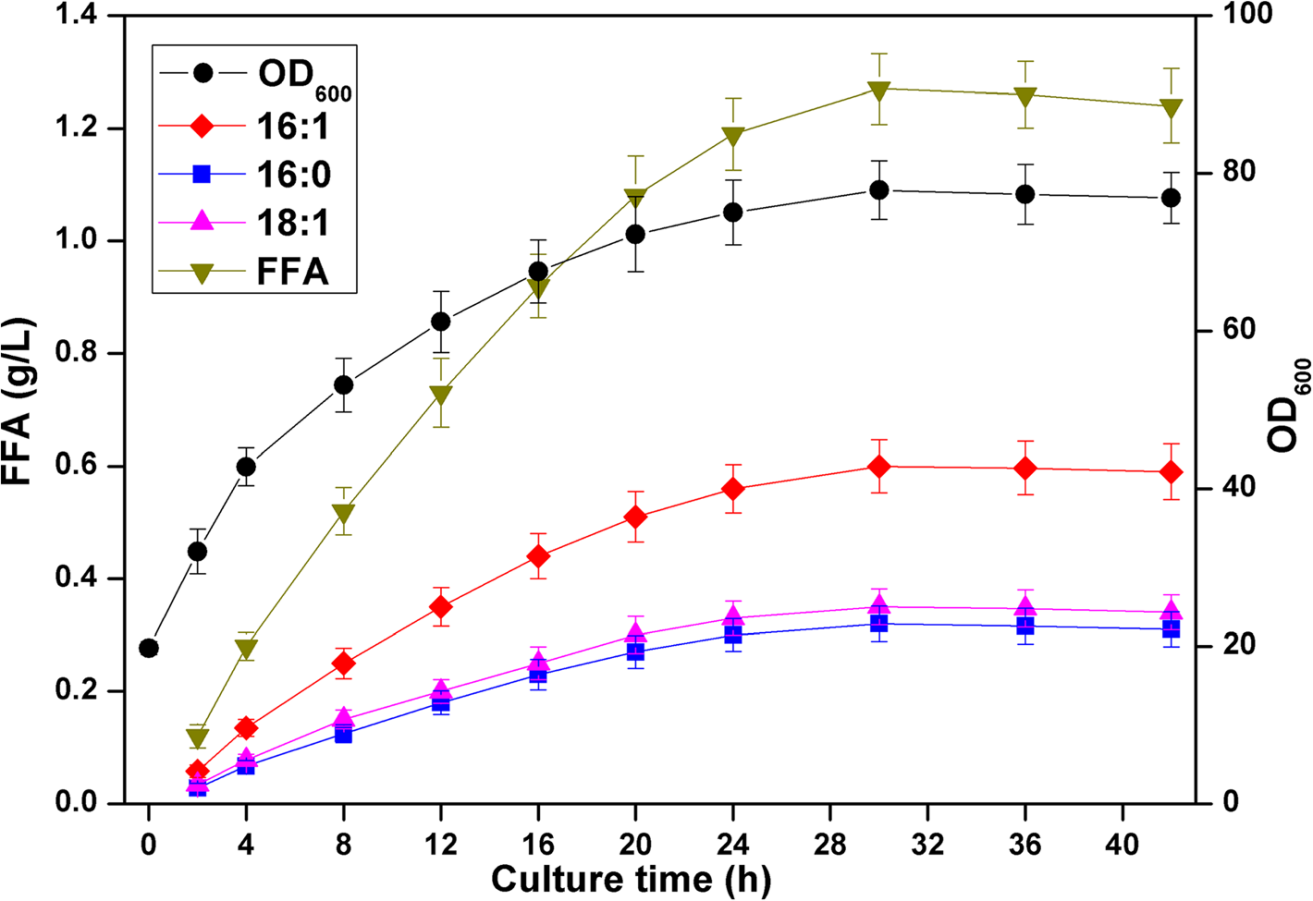
**Time profiles for cell density (OD**_**600**_**), palmitoleate (16:1Δ9), palmitate (16:0) and *cis*-vaccenate (18:1Δ11) and total free fatty-acid production during fed-batch fermentation of the finally engineered strain BL21ΔfadD/pE-AtFatAssi2&pA-acc.** FFA, free fatty acids; OD, optical density.

To further investigate the feasibility for larger-scale production of free MUFAs, the finally engineered strain BL21ΔfadD/pE-AtFatAssi2&pA-acc was cultured in a 5-L-scale laboratory fermenter. Cell density, residual glucose, and FFA production were monitored over the course of the experiment. The residual glucose was maintained at a very low level (below 1 g/L) throughout the whole fermentation process. Figure 6 shows the time profiles for cell density, and concentrations of different types of FFA for 42-h fed-batch fermentations. For approximately 30 h post-induction, the bacteria grew very fast to an OD_600_ of 80 or so. FFAs accumulated rapidly in the culture broth. The highest FFA production was obtained after being induced for 30 h, that is, 1.27 g/L, corresponding to a volumetric productivity of 5.4 mg/(L · h · OD_600_). The titers of palmitoleate, palmitate and *cis*-vaccenate increased gradually along with the bacterial cell growth, and the ratios of these three fatty acids remained stable. The two types of UFA, palmitoleate and *cis*-vaccenate, made up of more than 75% of the total FFA profiles. The above results obtained under the fermentor level demonstrated that this engineered *E. coli* strain had the potential to produce free MUFAs on a large scale. Compared with many other fatty-acid-producing systems [42,57], the final titer of FFAs was not very high and its industrial application was still far from occurring. However, the fatty-acid composition was greatly improved as SFAs always made up more than 60% of the total fatty-acid profiles in previous strains [20,42,57].

## Conclusions

In this study, we have successfully constructed an engineered *E. coli* strain capable of producing high levels of free MUFAs. By introducing four distinct genetic variations targeted at fatty-acid production and saturation-level regulation, the finally engineered strain produced 1.27 g/L of FFAs and the major proportions of these FFAs were MUFAs (palmitoleate and *cis*-vaccenate). Considering both the excellent oxidative stability and cold-flow properties of biodiesel derived from MUFAs, the fuel manufactured by the current *E. coli* strain would be more suitable for diesel engines. In addition, the results of this work would give some implications to improve the quality of biodiesel from higher plants and microalgae.

## Methods

### Bacterial strains

ΔA list of bacterial strains and recombinant plasmids used in this study is presented in Table 4. The one-step gene inactivation strategy, previously described by Datsenko and Wanner [58], was applied to knock out the chromosomal genes in *E. coli* BL21 star(DE3). Oligonucleotide primers used for gene disruption are listed (see Additional file 3: Table S1). For the construction of strain BL21ΔfadD, a linear DNA fragment containing the FRT-flanked kanamycin resistance cassette was amplified with primers fadD_Del_F and fadD_Del_R from plasmid pKD4. The obtained disrupting fragments were electrotransformed into *E. coli* competent cells that carried the Red recombinase expression vector pKD46 and integrated into its chromosome. Successfully disrupted colonies were then transformed with plasmid pCP20 and induced at 42°C to eliminate the kanamycin resistance. PCR verifications were performed using primer pairs designed according to the sequences up- and downstream of disrupted regions (fadD_DelIden_F and fadD_DelIden_R).

**Table 4:** Strains and plasmids used in this study

**Strains or plasmids**	**Genotype/description**	**Sources**
Strains		
*E. coli* BL21 star(DE3)	*F*^*−*^*ompT hsdS*_*B*_ (r_B_^−^ m_B_^−^) *gal dcm rne131* (DE3)	Invitrogen
*E. coli* BL21 star(DE3) ΔfadD	Knockout of *fadD* encoding acyl-CoA synthetase	This study
Plasmids		
pACYCduet-1	*Cm*^*r*^*oriP15A lacI*^*q*^*T7p*	Novagen
pET30a	*Kan*^*r*^*oripBR322 lacI*^*q*^*T7p*	Novagen
pEASY-Blunt	*Kan*^*r*^*Amp*^*r*^*oripUC*	Transgen
pE-ssi2	pET30a harboring *Arabidopsis* fatty-acid desaturase	[59]
pA-acc	pACYCduet-1 harboring *E. coli* acetyl-CoA carboxylase	[60]
PEASY-AtFatA	pEASY-Blunt harboring *Arabidopsis* thioesterase	This study
pA-AtFatA	pACYCduet-1 harboring *Arabidopsis* thioesterase	This study
pE-AtFatAssi2	pET30a harboring *Arabidopsis* fatty-acid desaturase and thioesterase	This study
pKD46	*Ap*^*r*^*oriR101 repA*(Ts) *λ Red* (*γ*, *β* and *exo*)	Coli Genetic Stock Center
pKD4	*FRT-Kan*^*r*^*-FRT oriR6K*	Coli Genetic Stock Center
pCP20	*Ap*^*r*^*Cm*^*r*^*repA*(Ts) *FLP*	Coli Genetic Stock Center

### Plasmids construction

The fatty-acid desaturase from *A. thaliana* (*ssi2*) was cloned to the expression vector pET30a resulting plasmid pE-ssi2 in a previous study [59]. The four subunits of native *E. coli* acetyl-CoA carboxylase were also cloned into a single expression vector pACYCduet-1, resulting pA-acc (see Additional file 4: Figure S3) in another study [60]. The *Arabidopsis* fatty acyl-ACP thioesterase (*AtFatA*) [AK176105: GenBank] gene was amplified using cDNA of *Arabidopsis* as a template and with primers AtFatA_F_NcoI and AtFatA_R_SalI containing the start and stop codons as well as the restriction sites of *Nco*I and *Sal*I. The amplified PCR products were analyzed by agarose gel electrophoresis and directly ligated into the pEASY-Blunt vector (Transgen, Beijing, China), resulting pEASY-AtFatA. The ligation products were transformed into *E. coli* DH5a-competent cells by heat-pulse transformation, and the antibiotic resistant transformants were selected to sequence *AtFatA*. The plasmid pEASY-AtFatA was double-digested with *Nco*I and *Sal*I, and the digested AtFatA fragment was withdrawn and ligated to pACYCduet-1 expression vector predigested with the same restriction enzymes and generated pA-AtFatA. PCR reaction was performed using pA-AtFatA as a template and a primer pair that allowed the amplification of the T7 promoter sequence along with the AtFatA structural gene. The PCR product, T7AtFatA was then cloned into pE-ssi2 between *Sal*I and *Not*I sites, to create pE-AtFatAssi2 (see Additional file 5: Figure S4). Successful gene cloning was verified by colony PCR, restriction mapping and direct nucleotide sequencing.

### Protein expression and gel electrophoresis analysis

Single colonies of *E. coli* strains harboring different recombinant plasmids were used to inoculate liquid LB medium containing appropriate antibiotics and grown overnight at 37°C. The saturated culture was diluted 1:100 in fresh LB medium and incubated under the same conditions. When the OD_600_ reached about 0.6, IPTG was added to a final concentration of 0.1 mM, and cell growth was continued for 4 h. The cells pelleted from 5 ml of culture were suspended in Tris-HCl buffer (pH 8.0) and subjected to ultrasonication. The mixture was centrifuged and the supernatant obtained was mixed with 2× sodium dodecyl sulfate (SDS) sample buffer, heated at 100°C for 10 minutes and then analyzed by SDS-PAGE.

### Shake-flask level cultivation

Shake-flask experiments were carried out in triplicate series of 250-ml Erlenmeyer flasks containing 50 ml of M9 minimal medium (6 g/L Na_2_HPO_4_, 3 g/L KH_2_PO_4_, 1 g/L NH_4_Cl and 0.5 g/L NaCl) supplemented with 1 mM MgSO_4_ and 20 g/L glucose as the carbon source. *E. coli* strains harboring different recombinant plasmids were inoculated to the culture medium and incubated in a gyratory shaker incubator at 37°C and 200 rpm. When the OD_600_ of the culture reached about 0.6, IPTG was added to a final concentration of 0.1 mM to induce recombinant protein expression and FFA production. The culture temperature was then switched to 30°C. Cell density, residual glucose and FFA production were measured during the whole fermentation courses.

### Fermentor scale cultivation

For large-scale production of free MUFAs, fed-batch cultivation was carried out in a Biostat B plus MO5L fermentor (Sartorius, Göttingen, Germany) containing 3 L of growth medium (3 g/L (NH_4_)_2_SO_4_, 1 g/L citrate, 1 g/L citrate sodium, 1.5 g/L KH_2_PO_4_, 1.9 g/L KCl, 75.6 mg/L FeSO_4_) that was sterilized at 115°C for 30 minutes. Glucose (20 g/L), MgSO_4_ (3 g/L) and trace elements (1 ml/L, 3.7 g/L (NH_4_)_6_Mo_7_O_24_ · 4H_2_O, 2.9 g/L ZnSO_4_ · 7H_2_O, 24.7 g/L H_3_BO_3_, 2.5 g/L CuSO_4_ · 5H_2_O, 15.8 g/L MnCl_2_ · 4H_2_O) were autoclaved or filter-sterilized separately and added prior to initiation of the fermentation. Then, 50 ml of inoculum was prepared by incubating the culture in shake flasks containing liquid LB medium overnight at 37°C. The fermentation was first operated in a batch mode and the control settings were: 37°C, stirring speed 600 rpm, and airflow 2 L/minute. During the fermentation process, the pH was controlled at 7.0 via automated addition of ammonia. Antifoam 204 was added to prohibit foam development. The agitation was associated with the dissolved oxygen (DO) to maintain a DO concentration above 20% saturation. After the initial glucose was nearly exhausted, fed-batch mode was commenced by feeding a solution containing 50% of glucose at appropriate rates and the residual glucose was maintained at a very low level. When the cells were grown to an OD_600_ of about 20, IPTG was added to the culture at a concentration of 0.5 mM and the culture temperature was switched to 30°C. Samples of fermentation broth were taken at appropriate intervals to determine cell density and FFA production.

### Free fatty-acid extraction and fatty-acid methyl esters preparation

FFAs were extracted from the culture broth following the procedure of Steen *et al*. [14] with some modifications. 0.4 ml of HCl and 4 ml of ethyl acetate were added to 4 ml of culture broth, spiked with 10 mg/L of arachidate as an internal standard. The mixture was vortexed for 30 s followed by shaking at 200 rpm for 30 minutes. The organic layer was separated and a second extraction was performed by the addition of another 4 ml of ethyl acetate.

Fatty acid methyl esters (FAMEs) were prepared according a standard procedure [61]. FFAs extracted in the ethyl acetate phase were evaporated to dryness under a stream of nitrogen, then suspended in 3 ml of boron trifluoride/methanol (1:4, by volume) and heated at 70°C for 30 minutes in sealed tubes. Esterified fatty acids were extracted by addition of 3 ml of hexane.

### Analytical methods

Cell density was determined by measuring the absorbance of the culture broth at 600 nm (an OD_600_ of 1.0 corresponds to 0.43 g dry cell weight per liter). The residual glucose in the culture medium was quantified using an SBA-40D Biological Sensing Analyzer (Biology Institute of Shandong Academy of Sciences, China).

FAMEs were identified using an Agilent (Santa Clara, United States) 7890A GC coupled to an Agilent 5975C quadrupole mass detector. The GC-MS conditions were as follows: a 30-m HP-INNOWax column (internal diameter 0.25 mm, film thickness 0.25 μm); an oven temperature program composed of an initial hold at 100°C for 5 minutes, ramping at 10°C per minute to 250°C, and a final hold at 250°C for 3 minutes; high-purity nitrogen as carrier gas with a linear velocity of 1 ml/minute; an ion source temperature of 220°C and EI ionization at 70 eV.

FAMEs contents were determined by a Varian (Palo Alto, United States) GC-450 system equipped with a 30 m HP-5 column (internal diameter 0.32 mm, film thickness 0.25 μm) and using high-purity nitrogen as carrier gas with a linear velocity of 1 ml/minute. The injector temperature was 250°C, the FID temperature was 300°C and the split ratio was 1:10. The oven was initially set at 100°C for 5 minutes, increased at 20°C per minute to 160°C, then ramped at 10°C per minute to 250°C and finally held at 250°C for 3 minutes.

## Abbreviations

ACCase: Acetyl-CoA carboxylase; ACP: acyl carrier protein; AtFatA: gene encoding a FatA-type thioesterase from *Arabidopsis*; DO: dissolved oxygen; DW: dry weight; FAME: fatty-acid methyl esters; FAS: fatty acid synthase; FFA: free fatty acid; FID: flame ionization detector; GC-MS: gas chromatography-mass spectrometry; IPTG: isopropyl-ß-D-thiogalactopyranoside; MUFA: monounsaturated fatty acid; OD: optical density; PFUA: polyunsaturated fatty acid; SFA: saturated fatty acid; ssi2: gene encoding a fatty-acid desaturase from *Arabidopsis*; UFA: unsaturated fatty acid.

## Competing interests

The authors declare that they have no competing interests.

## Authors’ contributions

YC: conception and design, data collection and analysis, manuscript writing and final approval of the manuscript. WL: data collection and analysis, critical revision and final approval of the manuscript. XX: data collection, critical revision and final approval of the manuscript. HZ: data collection, critical revision and final approval of the manuscript. JW: data analysis, critical revision and final approval of the manuscript. MX: conception and design, financial support, manuscript writing and final approval of manuscript. All authors read and approved the final manuscript.

## Supplementary Material

Additional file 1: Figure S1 Identification of the *fadD* knockout *E. coli* strains. PCR verifications were performed with primers fadD_DelIden_F and fadD_DelIden_R (Additional file 3: Table S1) corresponding to sequences up- and downstream of the disrupted regions. Lane M, DNA molecular weight markers; lane 1, strain BL21/ΔfadD by disrupting the *fadD* gene; lane 2 the original strain BL21 star(DE3). Click here for file

Additional file 2: Figure S2 GC chromatogram of free fatty-acid methyl esters from different metabolically engineered *E. coli* strains. **A**, BL21/pA-AtFatA; **B**, BL21/pE-AtFatAssi2; **C**, BL21ΔfadD/pE-AtFatAssi2; **D**, BL21ΔfadD/pE-AtFatAssi2&pA-acc. Click here for file

Additional file 3: Table S1 Primers used in this study for gene disruption, verification and plasmids construction. Click here for file

Additional file 4: Figure S3 The recombinant plasmid pA-acc overexpressing the four subunits of native *E. coli* acetyl-CoA carboxylase. Click here for file

Additional file 5: Figure S4 The recombinant plasmid pE-AtFatAssi2 coexpressing the acyl-ACP thioesterase AtFatA and the fatty-acid desaturase SSI2. Click here for file
