# Supplementary figures and images for: Production of free monounsaturated fatty acids by metabolically engineered Escherichia coli
Source: Biotechnol Biofuels. 2014 Apr 10;7:59. doi: 10.1186/1754-6834-7-59 (PMC4021618; doi:10.1186/1754-6834-7-59)

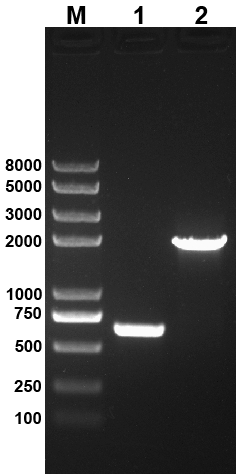

Supplement: Additional file 1: Figure S1 — Identification of the fadD knockout E. coli strains. PCR verifications were performed with primers fadD_DelIden_F and fadD_DelIden_R (Additional file 3: Table S1) corresponding to sequences up- and downstream of the disrupted regions. Lane M, DNA molecular weight markers; lane 1, strain BL21/ΔfadD by disrupting the fadD gene; lane 2 the original strain BL21 star(DE3). [file 1754-6834-7-59-S1.tiff]

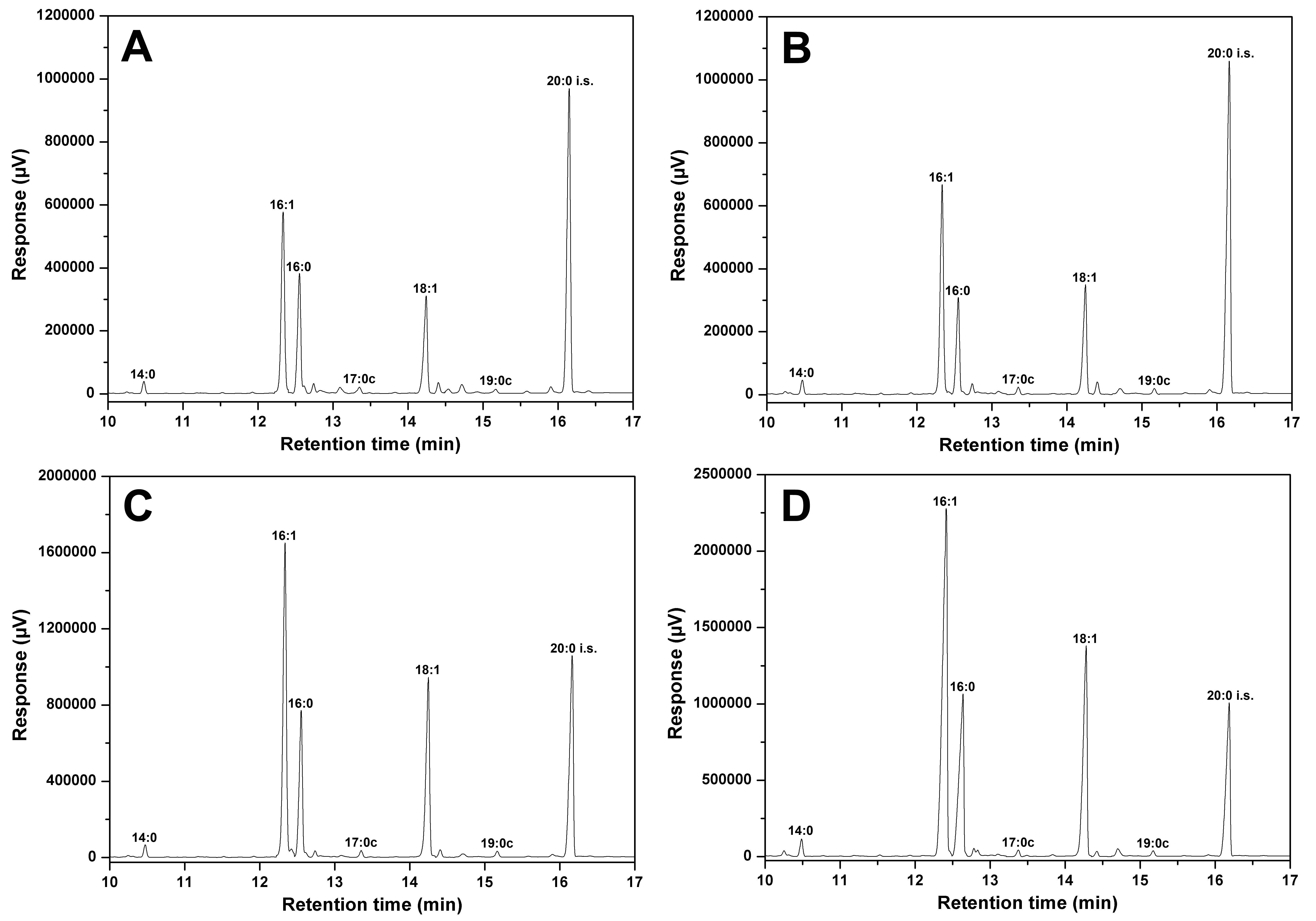

Supplement: Additional file 2: Figure S2 — GC chromatogram of free fatty-acid methyl esters from different metabolically engineered E. coli strains. A, BL21/pA-AtFatA; B, BL21/pE-AtFatAssi2; C, BL21ΔfadD/pE-AtFatAssi2; D, BL21ΔfadD/pE-AtFatAssi2&pA-acc. [file 1754-6834-7-59-S2.tiff]

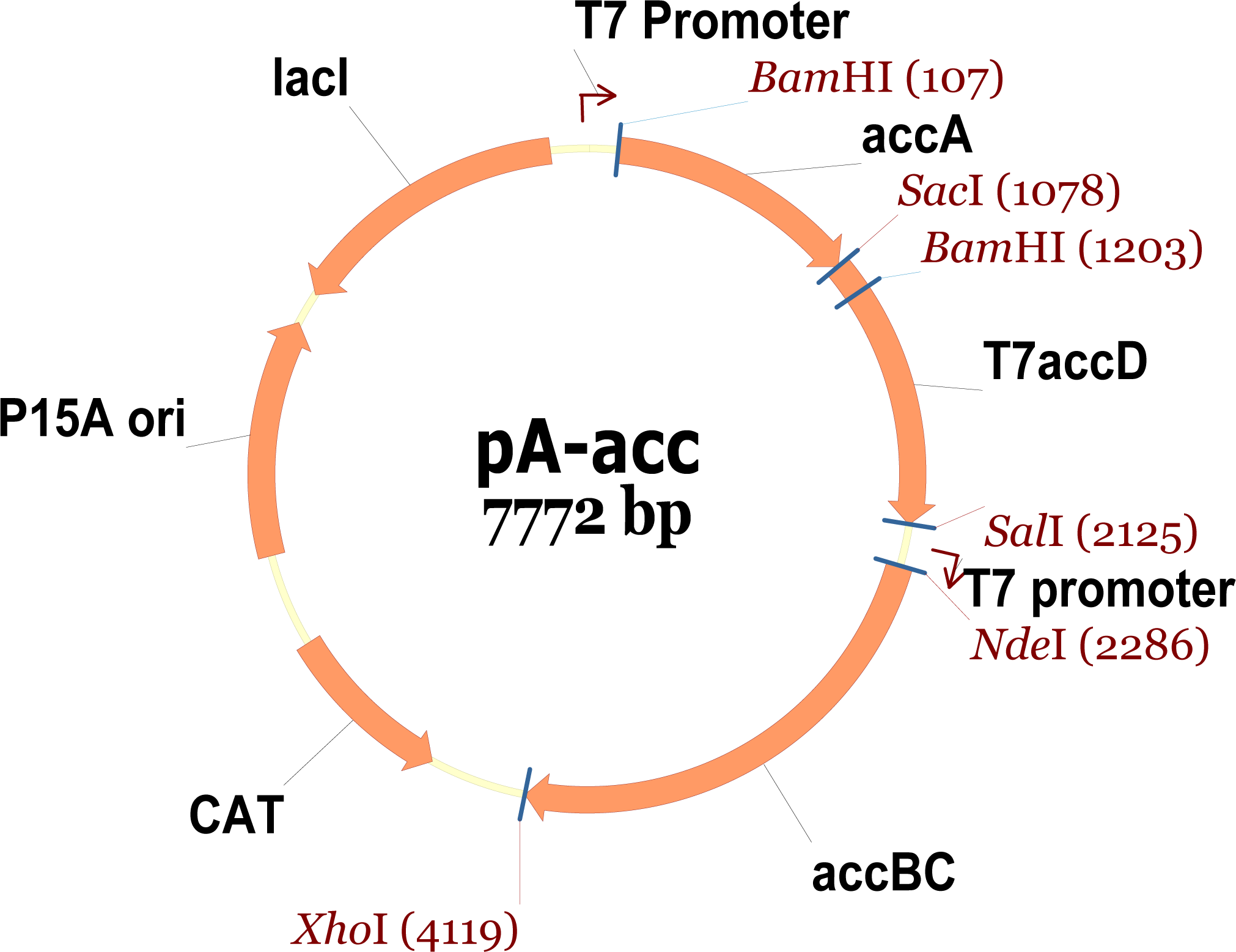

Supplement: Additional file 4: Figure S3 — The recombinant plasmid pA-acc overexpressing the four subunits of native E. coli acetyl-CoA carboxylase. [file 1754-6834-7-59-S4.tiff]

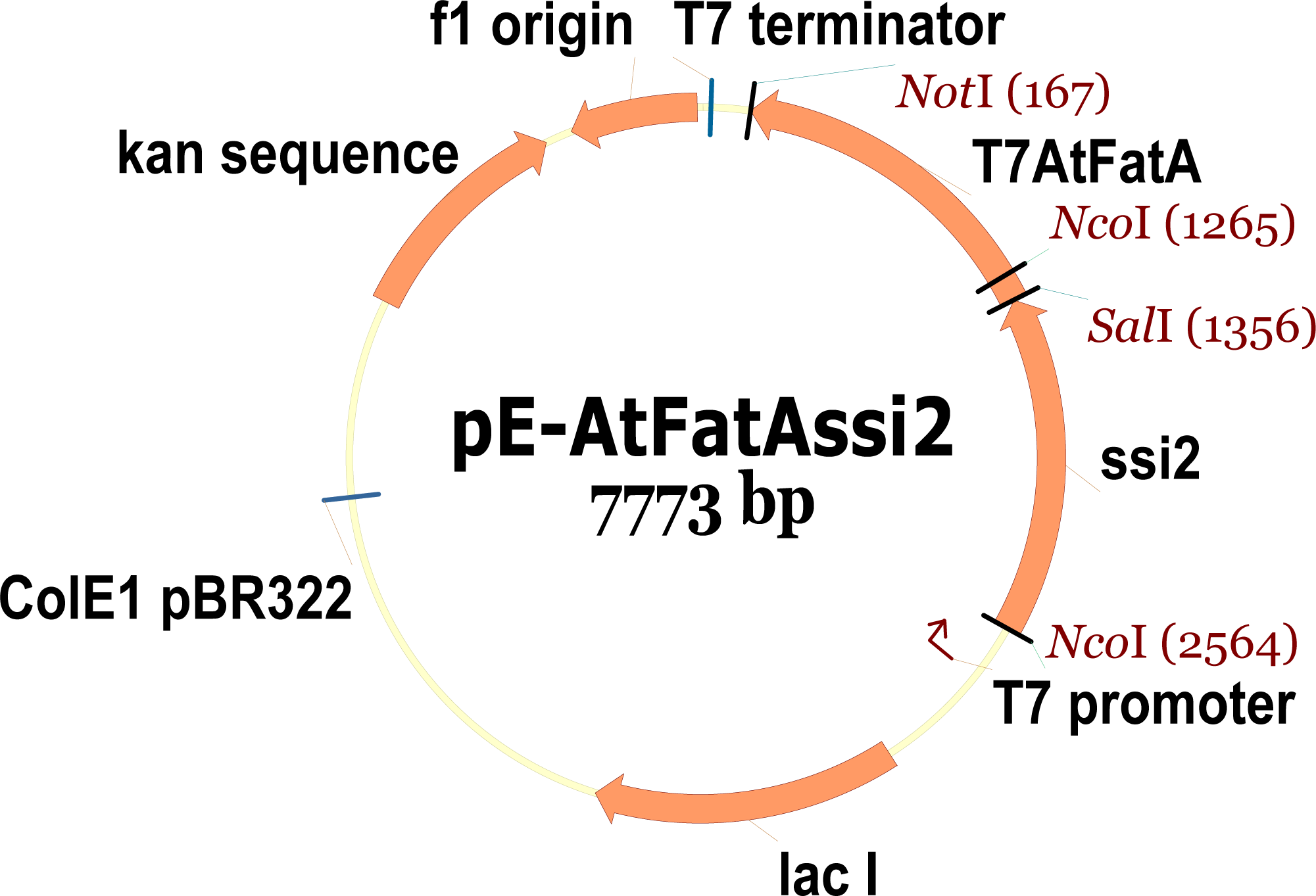

Supplement: Additional file 5: Figure S4 — The recombinant plasmid pE-AtFatAssi2 coexpressing the acyl-ACP thioesterase AtFatA and the fatty-acid desaturase SSI2. [file 1754-6834-7-59-S5.tiff]
