# Supplementary material for: Production of free monounsaturated fatty acids by metabolically engineered Escherichia coli
Source: Biotechnol Biofuels. 2014 Apr 10;7:59. doi: 10.1186/1754-6834-7-59 (PMC4021618; doi:10.1186/1754-6834-7-59)
Supplement: Additional file 3: Table S1 — Primers used in this study for gene disruption, verification and plasmids construction. [file 1754-6834-7-59-S3.doc]

**Table S1** Primers used in this study for gene disruption, verification and plasmids construction.

| Oligonucleotide primers | Sequences |
| --- | --- |
| fadD_Del_F | ATGTTTGAGCAGTCGGTCGCGCGCTACGCCGATCAACCTGCGTTTGTGAAgtgtaggctggagctgcttc |
| fadD_Del_R | TCAGGCTTTATTGTCCACTTTGCCGCGCGCTTCGTCACGTAATTCTCGTCatgggaattagccatggtcc |
| fadD_DelIden_F | CACTGCCTGCATGGAGAAAT |
| fadD_DelIden_R | TTGCGATGACGACGAACACG |
| AtFatA_F_NcoI | CATGCCATGGTTATGTTGAAGCTTTCGTGT |
| AtFatA_F_SalI | ACGCGTCGACTTAACTTGAAGGCTTCTTTC |
| T7AtFatA_F_SalI | ACGCGTCGACTAATACGACTCACTATAGGGG |
| T7AtFatA_F_NotI | AAGGAAAAAAGCGGCCGCTTAACTTGAAGGCTTCTTTCTC |
